# The History of Dentistry

**Published:** 1899-05

**Authors:** 


### THE HISTORY OF DENTISTRY.

Attention is called to the notice of the chairman of the committee appointed to report a "measure looking to the preparation of a full history of the dental profession."

The preparation of such a history will be a very serious task, and every year allowed to lapse increases the difficulty of its accomplishment. If it were confined solely to a history of dentistry in the United States and Canada, it would be a matter of great labor and conscientious research; but should it be proposed to extend this to a history of dentistry throughout the world, it would become a task which, it is feared, would be impossible of accomplishment. If such a history is attempted, it should be confined to this country, and there is quite sufficient of interest here to occupy the historian for a long period.

Several attempts have been made in this direction, the most valuable being "A History of Dental and Oral Science in America. Prepared under the direction of the American Academy of Dental Science, Boston, 1876;" but all are, at present, defective, and oftentimes lead the searcher after facts astray. What is needed is a condensed compilation of absolute and verified statements of historical facts. These may be difficult to procure, but there are many yet living who have passed through a large portion of the work of the last half of the present century, who could give valuable aid in this direction. To these the circular should appeal with force. An early answer to the queries will aid the committee in making up its report for the next meeting of the National Association.

